# Public Awareness of Triage Systems and Waiting Times During Emergency Department Visits in the Eastern Province, Saudi Arabia

**DOI:** 10.7759/cureus.51988

**Published:** 2024-01-10

**Authors:** Salahulddin Abuljadail, Hassan Alhussain, Yousef A. Alhamaid, Musawi Altaha, Mohammed Alhulayyil, Raed Alfayez, Abdullah Alhashim

**Affiliations:** 1 Orthopedic Surgery, College of Medicine, King Faisal University, Hofuf, SAU; 2 Family and Community Medicine, College of Medicine, King Faisal University, Hofuf, SAU; 3 Medicine, College of Medicine, King Faisal University, Hofuf, SAU; 4 Orthopedics, College of Medicine, King Faisal University, Hofuf, SAU

**Keywords:** waiting times, triage system, triage knowledge, ed utilization, emergency department

## Abstract

Introduction: The effective functioning of emergency departments (EDs) is essential for providing timely and appropriate medical care to patients with acute health issues. Triage, a critical aspect of ED operations, involves prioritizing patients based on the severity of their conditions. However, patients' understanding of the triage system plays a significant role in ensuring its efficient utilization. This study aims to examine the community's understanding of the triage system and the influencing factors.

Methodology: A cross-sectional study included 775 participants from the Eastern region of Saudi Arabia, all of whom had prior visits to the ED during their lifetime. The data was randomly collected between June and July 2023 through a self-administered online questionnaire.

Results: The results showed that a substantial number of participants (73.8%) were aware of why some patients are prioritized over others in terms of room allocation, while 26.2% lacked this awareness. Among those aware, the majority (80.5%) believed that the priority system is fair, while 19.5% disagreed. Roughly two-thirds (64.8%) of the participants understood the concept of triage, while 35.2% lacked knowledge in this regard. The history of ED visits, age, and highest level of education were significantly associated with knowledge (p < 0.001). Participants who had a history of ED visits, were aged 20-29 years, and had a bachelor's degree had the highest percentage of adequate knowledge. The study also found that the most common reasons for non-urgent visits to the ED were the unavailability of appointments elsewhere (35%) and the perception that the ED provides faster care (30.4%).

Conclusion: The study's assessment of triage knowledge reveals a moderate understanding among participants, with a majority demonstrating awareness of the prioritization system. The associations identified between demographic factors and triage knowledge highlight the importance of tailoring educational initiatives to specific groups. Individuals who visited the ED frequently and those who sought prior care exhibited better triage knowledge, suggesting potential opportunities for targeted interventions.

## Introduction

Emergency departments (EDs) are being visited more frequently nowadays than in previous years in several societies. Therefore, it is alleged that there are significant delays in delivering care to ED patients. Longer waiting times are correlated with poor patient satisfaction; however, improved awareness of the triage system improves satisfaction. Non-urgent patients reported visiting the ED for a variety of reasons in Saudi Arabia, including the lack of a regular primary healthcare provider, convenience, easy access, and the belief that they would receive better care [1].

Triage's primary objective is to deliver urgent care to those who need it the most. There are many triage systems used in EDs for classifying patients according to the severity of their conditions. The original concept of triaging patients on the scene was developed in France in the early 1800s, which was based on categorizing injured patients on the battlefield [2]. Later in the early 1960s, the same concept has been implemented in hospital EDs due to the increasing number of cases and non-urgent conditions [3]. Saudi Arabia employs the widely used Canadian Triage and Acuity Scale (CTAS), which categorizes patients into one of five levels based on how urgent their conditions are. In level I (resuscitation), the physician must see the patient immediately because of conditions that impose direct threats to life mandating immediate saving interventions. In level II (emergent), the physician must see the patient within 15 minutes because of conditions that are a potential threat to life, limb, or function, requiring rapid medical intervention or delegated acts. In level III (urgent), the physician must address the patient within 30 minutes due to conditions that could potentially progress to a serious problem requiring emergency intervention. In level IV (less urgent), the physician can assess the patient within one hour due to conditions related to patient age, distress, or potential for deterioration or complications that would benefit from intervention or reassurance. In level V (non-urgent), the patient can be seen within two hours due to conditions that may be acute but non-urgent as well as conditions that may be part of a chronic problem with or without evidence of deterioration [4].

Understanding the triage system can help patients to have a greater sense of satisfaction with the medical service provided in the ED as well as improvement of the healthcare system. While several studies on this topic have been done around the world, our study focuses on the eastern region of Saudi Arabia. This study aims to evaluate public awareness of triage systems in patients attending the emergency department in the eastern region of Saudi Arabia.

## Materials and methods

A cross-sectional study was conducted from June to July 2023 among adults who visited the ED in the Eastern Province of Saudi Arabia. The sample size was 385, determined by OpenEpi®️ version 3.0 software. The margin of error was determined as 5%, confidence 95%, 3.9 million for population size, and 50% for response distribution. All ED visitors aged 14 years old and above were included in this study, and those who had a language barrier and refused to participate were excluded.

The sampling technique that was used in this research is the non-probability convenience sampling technique, which was conducted among ED visitors. Data was collected using a web-based, self-administered questionnaire, which included four sections: participants' sociodemographic data, history of emergency hospital visits, participants' knowledge about the triage system in the ED, and the perspective of the participants regarding their preferences and reasons for visiting ED. Additionally, Bloom's classification cutoff points for knowledge were used as follows: an appropriate knowledge score of 75%-100% was regarded adequate, a suitable knowledge score of 50%- 74% was considered satisfactory, and a score of less than 50% was considered poor knowledge. A pretested anonymous self-administered electronic survey was utilized after obtaining permission to use it [5].

Collected data was analyzed using the International Business Machines (IBM) Statistical Package for the Social Sciences (SPSS) version 27.0.1 (Armonk, NY). The analysis involved both descriptive and inferential statistical tests. Descriptive statistics were used to summarize and describe the characteristics of the study participants and the findings. Frequencies and percentages were calculated for categorical variables. For continuous variables such as knowledge scores, mean and standard deviation were calculated for normally distributed variables, while median and interquartile range (IQR) were calculated for non-normally distributed variables. The normality of data was checked using the Shapiro-Wilk test. All data gathered for this study remained confidential and exclusively served scientific research purposes, strictly following ethical standards for research involving human subjects. Ethical clearance to carry out this research was obtained from the ethical approval committee at King Faisal University (reference number: KFU-REC-2023-JUN-ETHICS1005) and complied with the Declaration of Helsinki. Participation in this research was entirely voluntary and optional, with informed consent provided on the initial page. The data analysis and publication procedures did not involve any identifiable personal information. Ethical approval for the study was obtained before data collection began.

## Results

The total collected sample was 775 participants. The participant demographics revealed a diverse group, with 61.8% females and 38.2% males. Age distribution showed 20-29 years as the largest group (46.8%), while 50 and above constituted 6.6%. Education-wise, 50.1% had a bachelor's degree, 17.5% were working in the health sector, and 18.2% were unemployed. Of the participants, 44.4% have health insurance. Marital status varied, with 52.5% single and 43% married. Income distribution shows 32.8% earning <5,000 Saudi Riyals and 20.4% exceeding 15,000 Saudi Riyals (Table 1).

**Table 1:** Demographic characteristics of participants

		Count	%
Gender	Female	479	61.8%
	Male	296	38.2%
Age	14-19	108	13.9%
	20-29	363	46.8%
	30-39	151	19.5%
	40-49	102	13.2%
	50 and above	51	6.6%
Highest level of education	Ignorant	5	0.6%
	Primary school	12	1.5%
	Intermediate school	38	4.9%
	Completed high school	203	26.2%
	Bachelor	388	50.1%
	Diploma	72	9.3%
	Master	33	4.3%
	PhD	20	2.6%
	Other	4	0.5%
Current job	Government sector (not health)	77	9.9%
	Health sector	136	17.5%
	Military sector	28	3.6%
	Other	254	32.8%
	Private sector	98	12.6%
	Private work	41	5.3%
	Unemployed	141	18.2%
Marital status	Divorced	28	3.6%
	Married	333	43%
	Single	407	52.5%
	Widowed	7	0.9%
Monthly family income (Saudi Riyal)	Less than 5,000	254	32.8%
	From 5,000 to 10,000	211	27.2%
	Between 10,000 and 15,000	152	19.6%
	More than 15,000	158	20.4%
Do you have a health insurance?	Yes	344	44.4%
	No	431	55.6%

A significant proportion (55.5%) of participants visited the ED more than once in the last year, while 30.7% visited only once, and 13.8% did not visit at all. Most cases (50.2%) had their symptoms started on the same day of the ED visit, while 33.3% within a week of the ED visit. Long-term chronic issues (8.3%) and symptoms extending beyond a week (8.3%) were also noted. Participants' roles varied: 49.9% were patients, 43.6% were watchers, 4.5% were friends, and 1.9% were coworkers. Prior to ED visit, 35.7% sought care at their primary health center, while 64.3% did not. Most visits took place in government hospitals (60.4%), followed by private hospitals (28.5%), military hospitals (6.8%), and university hospitals (4.3%). Cases were not initially classified as critical in the majority (54.1%), while 25.7% were, and 20.3% were unsure. Within the past six months, 57.2% visited the ED, while 42.8% did not. A notable portion lacked a primary care doctor (60.8%), 22.8% had one, and 16.4% were uncertain. A majority (51.5%) expressed willingness to consult a primary care doctor before ED visits, while 48.5% would not (Table 2).

**Table 2:** History of emergency hospital visits

		Count	%
Have you (as a patient or a companion) ever visited the ED of any hospital in the last year?	No	107	13.8%
	Yes, more than once	430	55.5%
	Yes, only once	238	30.7%
When did the problem at that time start?	In a week period	258	33.3%
	In more than a week	64	8.3%
	Long-term chronic problem	64	8.3%
	Same day	389	50.2%
Were you a patient waiting to be seen or a family member?	Coworker	15	1.9%
	Friend	35	4.5%
	Patient	387	49.9%
	Watcher	338	43.6%
Did you try to visit your primary healthcare center before coming to the ED?	No	498	64.3%
	Yes	277	35.7%
What kind of hospital did you go to?	Government hospital	468	60.4%
	Military hospital	53	6.8%
	Private hospital	221	28.5%
	University hospital	33	4.3%
In the ED, was the case initially classified as a critical case?	No	419	54.1%
	Yes	199	25.7%
	I don't know	157	20.3%
Did you visit the ED in the last six months?	Yes	443	57.2%
	No	332	42.8%
Do you have a primary care doctor or another health provider?	No	471	60.8%
	Yes	177	22.8%
	I don't know	127	16.4%
Would you try to reach a primary care doctor before going to the ED?	Yes	399	51.5%
	No	376	48.5%

A substantial number (73.8%) of participants were aware of why some patients are being prioritized over others in terms of room allocation. Conversely, 26.2% lacked this awareness. Among those aware, the majority (80.5%) believed that the priority system is fair, while 19.5% disagreed. Roughly two-thirds (64.8%) of participants understood the concept of triage, while 35.2% lacked knowledge in this regard.

Knowledge score

The median knowledge score was 3.00, indicating an adequate overall understanding. The interquartile range (IQR) was 1 (Q3-Q1), ranging from 2 to 3 (Table 3).

**Table 3:** Participants' knowledge about the triage system

		Count	%
Do you know why some patients are taken to a room before others even though they may not have waited as long?	No	203	26.2%
	Yes	572	73.8%
Do you think this (what is in the previous question) is fair?	No	151	19.5%
	Yes	624	80.5%
Do you know what triage means?	No	273	35.2%
	Yes	502	64.8%

Knowledge level

**Figure 1:**
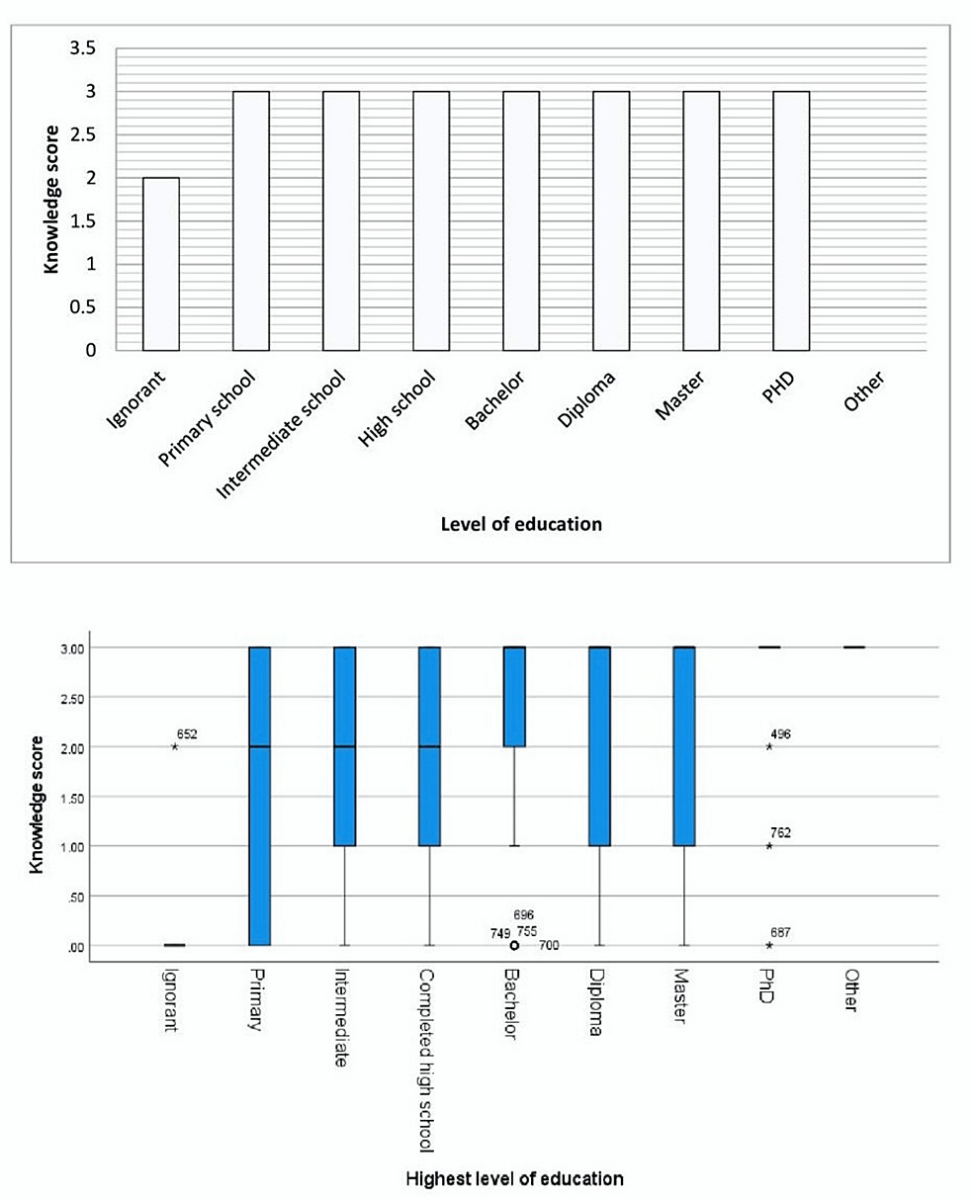
Distribution of knowledge scores with levels of education

Participants' knowledge levels were categorized as follows: adequate (76.5%), poor (11.9%), and satisfactory (11.6%) (Table 4 and Figure 1).

**Table 4:** Participants' knowledge about the triage system IQR: interquartile range

	Median		Maximum		Minimum		IQR (Q_3_-Q_1_)
Knowledge score	3.00		3.00		0.00		1 (3-2)
				Count		%	
Knowledge level		Adequate		593		76.5%	
		Poor		92		11.9%	
		Satisfactory		90		11.6%	

**Figure 2:**
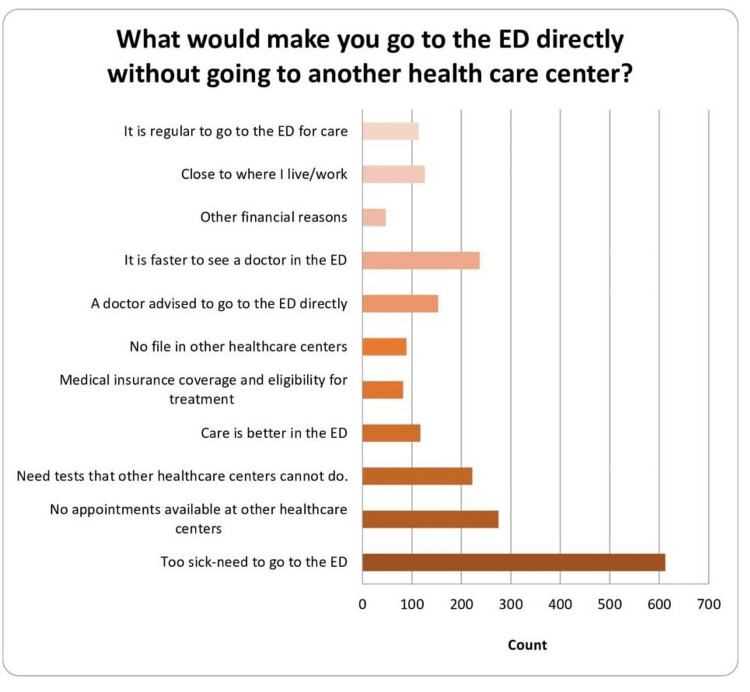
Causes of going to emergency directly without going to a health center ED: emergency department

Findings indicate that direct ED visits were primarily driven by severe illness (80%), lack of appointments elsewhere (35%), faster to see a doctor in the ED (30%), and the need for specialized tests (28.6%). Perceived superior care (15%), insurance considerations (10%), absence of records (11.4%), and professional advice (19.7%) also influence direct ED utilization (Figure 2).

**Figure 3:**
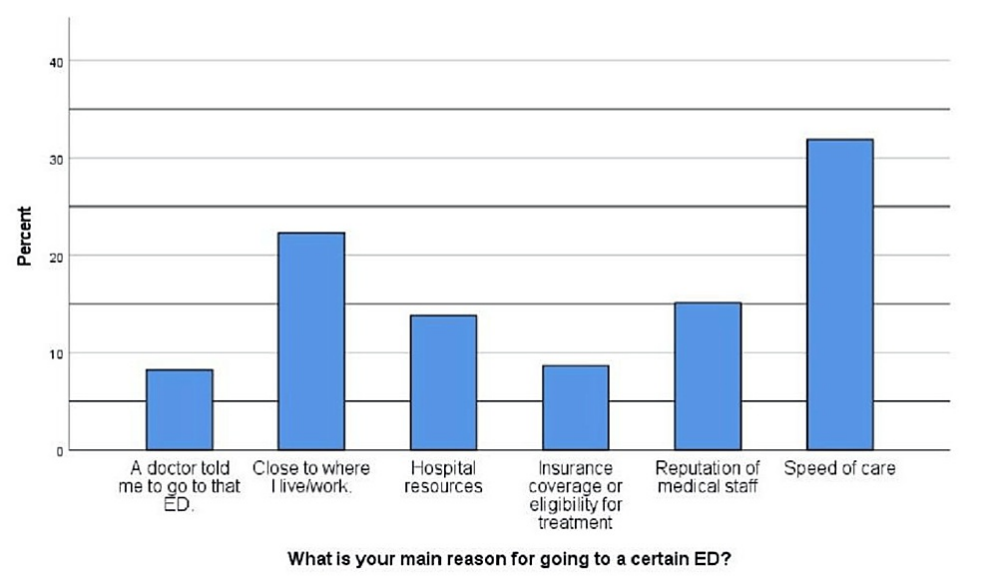
Reasons for selecting a specific emergency department ED: emergency department

Participants indicated their primary motivations for selecting a specific ED. Notable reasons included proximity to their residence or workplace (22.3%), the speed of care (31.9%), the reputation of the medical staff (15.1%), and hospital resources (13.8%). Additionally, insurance coverage or eligibility for treatment (8.6%) and a doctor's recommendation (8.3%) were cited as factors influencing their choice of ED visit (Figure 3).

For each demographic characteristic, Pearson Chi-square tests were conducted to examine the association with knowledge of the triage system. A Pearson Chi-square test yielded a non-significant result (χ² = 0.428, p = 0.807). The Chi-square test conducted for age showed a significant result (χ² = 7.191, p = <0.001). Participants in the age group 20-29 have the maximum number of adequate knowledge (47.6%). For the highest level of education, the Chi-square test revealed a significant association (χ² = 56.304, p < 0.001), where those who had bachelor's degrees had a maximum percentage of adequate knowledge (53.6%). A significant association was found between current job and knowledge of triage (χ² = 32.308, p = 0.001). Similarly, marital status showed a significant association (χ² = 21.609, p = 0.001), where those who were single had maximum triage knowledge. Monthly family income displayed a significant association with triage knowledge (χ² = 24.175, p < 0.001). The Chi-square test conducted for health insurance yielded a non-significant result (χ² = 0.170, p = 0.918) (Table 5).

**Table 5:** Association of demographic characteristics of participants with knowledge of triage

		Knowledge						p-value
		Adequate		Poor		Satisfactory		
		Count	%	Count	%	Count	%	
Gender	Female	369	62.2%	54	58.7%	56	62.2%	0.807
	Male	224	37.8%	38	41.3%	34	37.8%	
Age	14-19	77	13%	14	15.2%	17	18.9%	<0.001>
	20-29	282	47.6%	43	46.7%	38	42.2%	
	30-39	124	20.9%	13	14.1%	14	15.6%	
	40-49	74	12.5%	14	15.2%	14	15.6%	
	50 and more	36	6.1%	8	8.7%	7	7.8%	
Highest level of education	Bachelor	318	53.6%	34	37%	36	40%	<0.001>
	Completed high school	147	24.8%	26	28.3%	30	33.3%	
	Diploma	53	8.9%	12	13%	7	7.8%	
	Ignorant	1	0.2%	4	4.3%	0	0%	
	Intermediate school	20	3.4%	8	8.7%	10	11.1%	
	Master	24	4%	3	3.3%	6	6.7%	
	Other	4	0.7%	0	0%	0	0%	
	PhD	18	3%	1	1.1%	1	1.1%	
	Primary school	8	1.3%	4	4.3%	0	0%	
Current job	Government sector (not health)	60	10.1%	6	6.5%	11	12.2%	<0.001>
	Health sector	124	20.9%	4	4.3%	8	8.9%	
	Military sector	23	3.9%	3	3.3%	2	2.2%	
	Other	190	32%	31	33.7%	33	36.7%	
	Private sector	72	12.1%	18	19.6%	8	8.9%	
	Private work	29	4.9%	5	5.4%	7	7.8%	
	Unemployed	95	16%	25	27.2%	21	23.3%	
Marital status	Divorced	17	2.9%	4	4.3%	7	7.8%	<0.001>
	Married	265	44.7%	33	35.9%	35	38.9%	
	Single	309	52.1%	51	55.4%	47	52.2%	
	Widowed	2	0.3%	4	4.3%	1	1.1%	
Monthly family income (Saudi Riyal)	Between 10,000 and 15,000	134	22.6%	11	12%	7	7.8%	<0.001>
	From 5,000 to 10,000	163	27.5%	26	28.3%	22	24.4%	
	Less than 5,000	175	29.5%	42	45.7%	37	41.1%	
	More than 15,000	121	20.4%	13	14.1%	24	26.7%	
Do you have health insurance?	No	328	55.3%	53	57.6%	50	55.6%	0.918
	Yes	265	44.7%	39	42.4%	40	44.4%	

We employed Pearson Chi-square tests to examine the connection between participants' history of ED visits and their knowledge of the triage system. Key findings were tabulated (Table 6).

**Table 6:** Association of ED visit history with knowledge of triage ED: emergency department

		Knowledge						p-value
		Adequate		Poor		Satisfactory		
		Count	%	Count	%	Count	%	
Have you (as a patient or a companion) ever visited the ED of any hospital in the last year?	No	61	10.3%	29	31.5%	17	18.9%	0.001
	Yes, more than once	354	59.7%	29	31.5%	47	52.2%	
	Yes, only once	178	30%	34	37%	26	28.9%	
When did the problem at that time start?	In a week period	193	32.5%	31	33.7%	34	37.8%	0.214
	In more than a week	45	7.6%	12	13%	7	7.8%	
	Long-term chronic problem	49	8.3%	11	12%	4	4.4%	
	Same day	306	51.6%	38	41.3%	45	50%	
Were you a patient waiting to be seen or a family member?	Coworker	12	2%	2	2.2%	1	1.1%	0.072
	Friend	19	3.2%	8	8.7%	8	8.9%	
	Patient	306	51.6%	40	43.5%	41	45.6%	
	Watcher	256	43.2%	42	45.7%	40	44.4%	
Did you try to visit your primary healthcare center before coming to the ED?	No	367	61.9%	61	66.3%	70	77.8%	0.012
	Yes	226	38.1%	31	33.7%	20	22.2%	
What kind of hospital did you go to?	Government hospital	367	61.9%	47	51.1%	54	60%	0.236
	Military hospital	39	6.6%	9	9.8%	5	5.6%	
	Private hospital	164	27.7%	33	35.9%	24	26.7%	
	University hospital	23	3.9%	3	3.3%	7	7.8%	
In the ED, was the case initially classified as a critical case?	I don't know	113	19.1%	19	20.7%	25	27.8%	0.355
	No	324	54.6%	48	52.2%	47	52.2%	
	Yes	156	26.3%	25	27.2%	18	20%	
Did you visit ED in the last six months?	No	241	40.6%	46	50%	45	50%	0.083
	Yes	352	59.4%	46	50%	45	50%	
Do you have a primary care doctor or another health provider?	I don't know	95	16%	18	19.6%	14	15.6%	0.049
	No	349	58.9%	57	62%	65	72.2%	
	Yes	149	25.1%	17	18.5%	11	12.2%	
Would you try to reach a primary care doctor before going to the ED?	No	258	43.5%	59	64.1%	59	65.6%	0.001
	Yes	335	56.5%	33	35.9%	31	34.4%	

The history of ED visits was significantly associated with knowledge (χ² = 40.866, p < 0.001). Notably, those who visited the ED more than once demonstrated better knowledge (59.7% adequate) compared to those who visited once (30% adequate) or not at all (10.3% adequate). No significant association was found between the timing of the problem and triage knowledge (χ² = 8.338, p = 0.214). The role at the ED showed a near-significant association with knowledge (χ² = 11.569, p = 0.072), with slight variations in knowledge across roles. The history of visiting a primary healthcare center before ED showed a significant association (χ² = 8.780, p = 0.012), indicating a connection between prior healthcare seeking and triage knowledge. The type of hospital visited exhibited no significant association with knowledge (χ² = 8.024, p = 0.236). Initial case classification as critical in the ED did not significantly associate with knowledge (χ² = 4.397, p = 0.355). Recent ED visits in the last six months showed a near-significant trend (χ² = 4.982, p = 0.083). Having a primary care doctor or another health provider significantly related to knowledge (χ² = 9.542, p = 0.049). The preference for reaching a primary care doctor before the ED displayed a significant association (χ² = 25.397, p < 0.001), suggesting that this preference is linked to triage knowledge.

## Discussion

The study presents a comprehensive analysis of the demographics, patterns of ED visits, participants' knowledge about the triage system, and factors influencing direct ED utilization in the Eastern Province of Saudi Arabia.

Regarding the demographics of participants, the majority of participants were females; this gender distribution could have implications for healthcare utilization patterns and awareness levels among different gender groups. The largest age group represented was individuals aged 20-29, suggesting that this age group may be more likely to visit EDs. Education and employment status indicate that a well-educated and healthcare-aware population was surveyed. A notable proportion of participants had health insurance, which may influence their healthcare-seeking behavior.

Over half of the participants visited the ED more than once in the last year, suggesting that some individuals rely on the ED for healthcare needs. A substantial number of cases had symptoms that started on the same day they visited the ED, emphasizing the need for immediate care in these cases. However, a significant portion (33.3%) presented within a week, indicating that not all cases were urgent. About one-third of participants sought care at their primary health center before visiting the ED, while the majority did not. This highlights the importance of promoting primary care utilization for non-urgent cases.

Governmental hospitals were the most common choice for ED visits, indicating a reliance on public healthcare facilities. A majority of cases were not initially classified as critical, potentially indicating a need for improved triage and assessment processes. A significant portion of participants lacked a primary care doctor, which could contribute to their reliance on ED services. A majority of participants expressed willingness to consult a primary care doctor before visiting the ED, indicating an opportunity to encourage primary care utilization.

The study found that a significant portion of participants were aware of why some patients are prioritized over others in terms of room allocation. This suggests that a considerable portion of the public in the Eastern Province of Saudi Arabia understands the concept of triage during ED visits [6,7].

The 26.2% of participants who lacked this awareness could represent a target group for educational interventions to improve their understanding of triage systems, potentially reducing misconceptions and enhancing the efficiency of ED utilization. This finding aligns with previous research conducted by Zachariasse et al. (2019), which reported a comparable level of public awareness regarding triage systems [8].

Among those aware of the priority system, a substantial majority believe that the system is fair. This positive perception is crucial as it suggests public trust in the ED's allocation of resources based on medical needs, consistent with the findings of Lauridsen (2020), who demonstrated a high level of public confidence in the fairness of triage systems in emergency care [9]. Approximately two-thirds of participants in the study demonstrated an understanding of the concept of triage. This suggests that a substantial portion of the population has a foundational knowledge of how patients are assessed and prioritized in the ED. This is in line with previous studies by Bazyar et al. (2019) and AlShatarat et al. (2022), indicating that public awareness and comprehension of triage systems are crucial components of effective emergency care delivery [10,11].

The study identified several factors influencing direct ED visits, with severe illness being the most significant driver. This highlights the crucial role of EDs in providing urgent care for serious medical conditions. Other factors such as the lack of appointments elsewhere, faster access to doctors, and the need for specialized tests also contribute to direct ED utilization. These findings resonate with the work of Uscher-Pines et al. (2013), who emphasized the role of perceived urgency and convenience in determining patient choices regarding ED visits [12]. The categorization of knowledge levels into adequate, poor, and satisfactory underscores the need for targeted educational efforts to address the knowledge gaps among those with poor or satisfactory levels of understanding. This study's categorization approach mirrors the findings of a study by von dem Knesebeck et al. (2022), highlighting the distribution of knowledge levels among the public in relation to emergency care [13].

Patients in the Eastern Province of Saudi Arabia consider several factors when choosing an ED. Notably, proximity to residence or workplace, speed of care, reputation of medical staff, and hospital resources were significant factors. Additionally, insurance coverage, as well as doctor recommendations, played a role in ED selection. These findings align with the research of Mosadeghrad (2019), who identified similar factors influencing ED choice, emphasizing the importance of location and quality of care perception [14].

Regarding the association of demographic characteristics with knowledge of triage, the age group 20-29 had the highest percentage of adequate knowledge. This suggests that younger individuals may have a better understanding of triage systems [15]. Those with a bachelor's degree had the maximum percentage of adequate knowledge, highlighting the role of education in enhancing awareness of triage [16]. A significant association between current job and triage knowledge was found, emphasizing the potential impact of occupation on awareness. Single individuals had the highest triage knowledge, indicating a potential link between marital status and awareness of healthcare processes. The income level was significantly associated with triage knowledge, suggesting that higher-income individuals may have better awareness.

The study investigated the relationship between a patient's history of ED visits and their knowledge of triage systems. A significant association was found, with those who visited the ED more than once demonstrating better knowledge. This finding corroborates the results of a study conducted by Johnson et al. (2018) in a similar healthcare setting, highlighting that repeated exposure to the ED may contribute to a deeper understanding of triage processes [17]. The timing of the problem did not show a significant association with triage knowledge, indicating that the awareness of triage systems is not strongly influenced by the timing of the medical issue. This result aligns with the findings of a previous study by Bijani et al. (2019), which also did not find a significant correlation between the timing of health issues and knowledge of ED triage [18].

Although the role of the visitor at the ED showed a near-significant association with knowledge, the variations in knowledge across roles suggest that certain roles within the ED may provide more exposure and understanding of triage systems. Similar findings were reported by Sherafat et al. (2019), emphasizing that healthcare professionals within the ED may have varying levels of familiarity with triage processes [19]. The history of visiting a primary healthcare center before the ED was significantly associated with triage knowledge, suggesting that prior health-seeking behavior is linked to a better understanding of triage. This result supports the conclusions of Bowden et al. (2020), who found that individuals with a history of primary care visits are more likely to have knowledge of healthcare systems, including triage [20].

The type of hospital visited did not exhibit a significant association with knowledge of triage systems, indicating that knowledge levels were not influenced by the choice of healthcare facility. This finding mirrors the observations made by Azizpour et al. (2022), suggesting that regardless of the hospital type, public knowledge of triage remains consistent [21]. The preference for reaching a primary care doctor before visiting the ED showed a significant association with triage knowledge, implying that individuals who prioritize primary care may be better informed about triage systems. This is in accordance with the study by Brennan et al. (1998), which emphasized the role of patient preferences in shaping their knowledge of healthcare processes [22].

While this research study provides valuable insights into the factors influencing direct ED visits and the knowledge levels of participants regarding the triage system, several limitations should be acknowledged. Firstly, the study's reliance on self-reported data introduces the potential for recall bias and inaccuracies in participant responses. Furthermore, the study's cross-sectional design limits the ability to establish causal relationships between variables. Lastly, the knowledge assessment may not fully capture participants' true understanding of the triage system, as it relies on a single score without considering the depth of their knowledge.

## Conclusions

Participants exhibit varying levels of knowledge about triage, with the majority having an adequate understanding. It is worth noting that sociodemographic characteristics such as age, education, employment status, marital status, and income are associated with differing levels of triage knowledge. This study underscores the importance of targeted educational interventions to enhance public awareness of the triage system and promote more informed healthcare choices among the population. Further research with larger and more diverse samples and longitudinal designs would provide a deeper understanding of these relationships and help refine healthcare policies and interventions.

## References

[1] Alyasin A, Douglas C (2014). Reasons for non-urgent presentations to the emergency department in Saudi Arabia. Int Emerg Nurs.

[2] Robertson-Steel I (2006). Evolution of triage systems. Emerg Med J.

[3] (2012). The Canadian Triage and Acuity Scale: Education Manual Module. https://caep.ca/wp-content/uploads/2017/06/module_1_slides_v2.5_2012.pdf.

[4] Beveridge R, Clarke BR, John S (1998). Implementation Guidelines for The Canadian Emergency Department Triage & Acuity Scale (CTAS). https://ctas-phctas.ca/wp-content/uploads/2018/05/ctased16_98.pdf.

[5] Qureshi NA (2010). Triage systems: a review of the literature with reference to Saudi Arabia. East Mediterr Health J.

[6] Alsulimani LK (2022). Public awareness of triage in emergency departments in Saudi Arabia in the era of COVID-19. Saud J Emerg Med.

[7] Alhabdan N, Alhusain F, Alharbi A, Alsadhan M, Hakami M, Masuadi E (2019). Exploring emergency department visits: factors influencing individuals' decisions, knowledge of triage systems and waiting times, and experiences during visits to a tertiary hospital in Saudi Arabia. Int J Emerg Med.

[8] Zachariasse JM, van der Hagen V, Seiger N, Mackway-Jones K, van Veen M, Moll HA (2019). Performance of triage systems in emergency care: a systematic review and meta-analysis. BMJ Open.

[9] Lauridsen S (2020). Emergency care, triage, and fairness. Bioethics.

[10] Bazyar J, Farrokhi M, Khankeh H (2019). Triage systems in mass casualty incidents and disasters: a review study with a worldwide approach. Open Access Maced J Med Sci.

[11] AlShatarat M, Rayan A, Eshah NF, Baqeas MH, Jaber MJ, ALashtawy M (2022). Triage knowledge and practice and associated factors among emergency department nurses. SAGE Open Nurs.

[12] Uscher-Pines L, Pines J, Kellermann A, Gillen E, Mehrotra A (2013). Deciding to visit the emergency department for non-urgent conditions: a systematic review of the literature. Am J Manag Care.

[13] von dem Knesebeck O, Koens S, Schäfer I, Strauß A, Klein J (2021). Public knowledge about emergency care-results of a population survey from Germany. Front Public Health.

[14] Mosadeghrad AM (2014). Factors influencing healthcare service quality. Int J Health Policy Manag.

[15] Altman MC (2021). A consequentialist argument for considering age in triage decisions during the coronavirus pandemic. Bioethics.

[16] Butler K, Anderson N, Jull A (2023). Evaluating the effects of triage education on triage accuracy within the emergency department: an integrative review. Int Emerg Nurs.

[17] Johnson KD, Gillespie GL, Vance K (2018). Effects of interruptions on triage process in emergency department: a prospective, observational study. J Nurs Care Qual.

[18] Bijani M, Khaleghi AA (2019). Challenges and barriers affecting the quality of triage in emergency departments: a qualitative study. Galen Med J.

[19] Sherafat A, Vaezi A, Vafaeenasab M, Ehrampoush M, Fallahzadeh H, Tavangar H (2019). Responsibility-evading performance: the experiences of healthcare staff about triage in emergency departments: a qualitative study. Iran J Nurs Midwifery Res.

[20] Bowden T, Lyell D, Coiera E (2020). Emergency care access to primary care records: an observational study. BMJ Health Care Inform.

[21] Azizpour I, Mehri S, Soola AH (2022). Disaster preparedness knowledge and its relationship with triage decision-making among hospital and pre-hospital emergency nurses - Ardabil, Iran. BMC Health Serv Res.

[22] Brennan PF, Strombom I (1998). Improving health care by understanding patient preferences: the role of computer technology. J Am Med Inform Assoc.

